# The research progress on radionuclides in osteoporosis diagnosis and drug efficacy monitoring

**DOI:** 10.3389/fphar.2025.1594903

**Published:** 2025-04-28

**Authors:** Jie Fu, Chi Zhang

**Affiliations:** ^1^ College of Future Technology, Peking University, Beijing, China; ^2^ Department of Orthopedics, Peking University International Hospital, Beijing, China

**Keywords:** osteoporosis, bone, radionuclides, diagnosis, efficacy monitoring

## Abstract

Osteoporosis is a common metabolic bone disease that seriously affects the quality of life and health of patients. Traditional diagnostic methods, such as dual energy X-ray absorptiometry (DXA), have limitations in early detection and dynamic monitoring, making it difficult to meet clinical needs. This paper focuses on the potential of radionuclide imaging techniques, such as Single Photon Emission Computed Tomography (SPECT) and Positron Emission Tomography (PET), in the early diagnosis of osteoporosis. The paper further elaborates on the importance of radionuclides in evaluating the therapeutic effect of osteoporosis drugs. By summarizing current research findings, this paper aims to emphasize the core role of radionuclides in the management of osteoporosis, and provide theoretical basis and practical guidance for optimizing the diagnosis and treatment strategies of bone metabolism diseases in the future.

## 1 Introduction

Osteoporosis is a common metabolic disease of the skeletal system, characterized by low bone mass and structural changes leading to increased bone fragility and increased risk of fractures. World Health Organization (WHO) reported that approximately 200 million people worldwide suffer from osteoporosis (Avenell et al., 2017). A study based on the 2019 Global Burden of Disease Study analysis showed that the number of cases of osteoporosis worldwide more than doubled between 1990 and 2019 (Zhu et al., 2023). In 2021, a meta-analysis showed that the global prevalence of osteoporosis was approximately 18.3% (approximately 23.1% for females, approximately 11.7% for males) (Salari et al., 2021). It is expected that the total number of global cases between 2030 and 2034 will reach 263.2 million, including 154.4 million females and 108.8 million males. The burden calculated based on disability adjusted life years (DALYs) is expected to reach 128.7 million, with 78.4 million females and 50.3 million males (Zhu et al., 2023).

With the aging of the global population and changes in lifestyle, osteoporosis has become one of the major public health issues in various countries and regions, whether in high human development index areas such as North America and Europe, or in areas with lower development indices. The prevalence of osteoporosis in the United States was 12.6% from 2017 to 2018 (Sarafrazi et al., 2021). From 2008 to 2018, the incidence rate of osteoporosis among people aged 50 and over in South Korea was 18.4 cases per 1,000 people per year (Das et al., 2024). The epidemiological survey in China in 2018 showed that the prevalence of osteoporosis among people aged 50 and over was 19.2%. Research shows that approximately 50% of women will experience osteoporotic fractures (Reid, 2020), while over 20% of men over the age of 50 will experience the same problem (Kanis et al., 2000). In the United States, there are approximately 1.5 million cases of fractures caused by osteoporosis each year (Salari et al., 2021), while in a meta-analysis in the Asia Pacific region, the overall fracture incidence rate was as high as 2000 cases per 100,000 people per year (Chandran et al., 2023). From this, it can be seen that in the face of the health threat of osteoporosis to the elderly population and the heavy economic burden on society and families, countries should pay more attention to public health policies, early screening, and intervention measures, and actively seek innovative and effective methods in the direction of early diagnosis and precise treatment.

## 2 Pathogenesis of osteoporosis

Normal bone remodeling includes processes of bone resorption and bone formation, where old bone is replaced with new bone by osteoclasts and osteoblasts, respectively. From childhood to early adulthood, the rate of bone formation exceeds the rate of bone resorption, and bone density reaches its peak in the third decade of life. Afterwards, balance shifts towards bone resorption, and bone density begins to slowly decrease. The main cause of osteoporosis is an imbalance in bone metabolism, with an increased ratio of bone resorption to bone formation, leading to progressive bone loss. In patients with osteoporosis, the degree of bone resorption is even greater than the rate of bone resorption during normal aging (Berger et al., 2008). The biological mechanisms of osteoporosis are complex and diverse, involving multiple levels of factors such as genetics, environment, and lifestyle, which interact and jointly determine an individual’s bone health.

### 2.1 Genetic factors

Genetic factors mainly affect bone size, bone mass, bone microstructure, and mechanical properties. Classic signal transduction theory provides a framework for understanding the operational mechanisms of key signaling pathways in bone metabolism. Signal transduction is the process of transmitting signals inside and outside the cell, which regulates cellular physiological functions through the interaction of receptors, signaling molecules, and effectors. The main bone metabolism signaling pathways identified by genome-wide association studies (GWAS) include the WNT signaling pathway, RANK signaling pathway, vitamin D signaling pathway, and estrogen signaling pathway (Zhang et al., 2024). They recognize signaling molecules through receptors, trigger intracellular signaling cascades, and affect gene expression and cell activity, jointly regulating osteoclasts and osteoblasts.

Wnt/β-catenin is a classic pathway in the Wnt signaling pathway, which is a lipid modified secreted glycoprotein. The gene expression of Wnt1 and Wnt7b can enhance the activity of the Wnt/β-catenin signaling pathway and promote bone formation (Lawson et al., 2022); Wnt3a can activate β-catenin signaling to inhibit osteoclast differentiation, and can also inhibit osteoclast differentiation by activating cAMP/PKA (Weivoda et al., 2016). It can be seen that Wnt protein plays an important dual role in osteoblast differentiation and osteoclast differentiation, and its abnormal activation or inhibition can lead to the occurrence of bone related diseases such as osteoporosis and delayed fracture healing.

The OPG-RANKL-RANK signaling pathway is an important pathway that regulates osteoclast function during bone remodeling. RANKL (RANK Ligand) is produced by osteoblasts and is a ligand that can directly bind to osteoclast progenitor cells (Lacey et al., 1998). It binds to RANK receptors on the surface of osteoclasts and their precursor cells, causing osteoclast precursor cells to differentiate into osteoclasts (Hsu et al., 1999). OPG (Osteoprotegerin) is a member of the TNF receptor superfamily and an effective inhibitor of osteoclastogenesis, which competitively inhibits the binding of RANKL and RANK, preventing premature maturation of osteoclasts and reducing bone resorption (Boyce and Xing, 2008).

Vitamin D binds to the vitamin D receptor (VDR) through its active form 1,25-dihydroxyvitamin D3, which is located on human chromosome 12 and contains 9 exons and 8 introns (Imamov et al., 2005). There are hundreds of genetic polymorphisms, among which Apa I, Bsm I, Taq I, and Fok I are enzyme cleavage sites closely related to bone density. As a nuclear transcription factor, VDR regulates RNA transcription and expresses biological functions. It is expressed on the cell surfaces of osteoblasts, osteoclasts, parathyroid glands, and kidneys, mediating the physiological metabolism of calcium and phosphorus. It can not only increase the production of osteocalcin and osteopontin, but also inhibit the proliferation and promote the differentiation of osteoclasts (Voltan et al., 2023).

Human estrogen receptors include two subtypes, ER α and ER β, located on chromosomes 6 and 14 respectively. They mediate ligand dependent transcription factors to regulate a range of bone metabolism activities (Khalid and Krum, 2016). Compared to ER β, ER α has a higher role in protecting bones and is currently the most studied effector factor (Nagandla and Thomas, 2024). After estrogen binds to ER expressed in osteoblasts, it stimulates the release of growth factors and increases the level of type I collagen mRNA in osteoblasts to promote bone remodeling; When estrogen binds to ER in osteoclasts, the activity of lysosomal enzymes is inhibited. Osteoclasts begin to apoptosis, and reduce bone resorption (Zhang et al., 2023).

In addition, there are many genes that have received more attention in recent years, such as lipoprotein receptor related protein 5 (LRP5), bone morphogenetic protein (BMP) gene, and Osterix (Osx) (Zhang, 2010). However, the relationship between many genetic variations and bone density exhibits heterogeneity across different populations, and can only explain 20% of the differences in human bone density (Zhu et al., 2021), suggesting that the explanatory power of genetic variations on bone density is limited, and there may also be complex genetic heterogeneity and polygenic models (Morris et al., 2019).

### 2.2 Environmental factors

In addition to the role of genetic inheritance, environmental factors involve various aspects such as hormone aging, lifestyle, and drug exposure (Yang et al., 2023). They affect the signaling pathways and related genes of bone metabolism. These changes lead to decreased bone mass and microstructural damage, resulting in decreased bone strength as shown in Table 1.

**TABLE 1:** Environmental etiology of primary osteoporosis.

Hormone aging	Estrogen deficiency causes bone loss
	Hyperparathyroidism accelerates bone loss
	Aging leads to a decrease in bone marrow mesenchymal stem cells and muscle mass
Life-style	Smoking and drinking lead to bone loss
	Exercise promotes bone formation and remodeling
	Calcium phosphorus balance is beneficial for maintaining peak bone mass and degree of bone mineralization
Drug exposure	Corticosteroids, gonadotropin-releasing hormone analogs, excessive thyroid hormones, *etc.*, Can reduce the number of osteoblasts

## 3 Diagnostic methods for osteoporosis

In clinical practice, for patients suspected of having osteoporosis, doctors will pay attention to their age, gender, medical history, and family history, and conduct preliminary screening based on clinical symptoms and signs, supplemented by laboratory tests (Zhang et al., 2022). These biochemical indicators themselves cannot be used to diagnose osteoporosis, but can be used for diagnostic classification and differential diagnosis of secondary osteoporosis. The key to diagnosis is to use imaging examinations to ultimately determine the presence of osteoporosis as shown in Table 2 (Cheng et al., 2020; Zhang et al., 2014).

**TABLE 2:** Clinical diagnostic methods and procedures for osteoporosis.

Clinical symptoms	Bone pain accompanied by muscle spasms, restricted movement, reduced height, spinal deformities, fragility fractures*etc.*	
Patient’s medical history	Inquire about age, gender, family history, current medical history, medication history*etc.*	
Laboratory Examination	Basic laboratory tests: blood routine, urine routine, liver function, kidney function, serum protein electrophoresis, blood calcium, 25 hydroxyvitamin D3, blood phosphorus, urine calcium, urine sodium	
	Bone conversion indicators: bone specific alkaline phosphatase, osteocalcin, C-terminal propeptide/N-terminal propeptide of type I procollagen, fasting 2-h urine calcium/creatinine ratio, tartrate resistant acid phosphatase, serum/urine C-terminal peptide cross-linking of type I collagen, urine N-terminal peptide cross-linking of type I collagen	
	Differential diagnostic indicators: erythrocyte sedimentation rate, C-reactive protein, six sex hormones, 1,25-dihydroxyvitamin D3, parathyroid hormone, thyroid function, free cortisol in urine, blood gas analysis, Bence-Jones protein, and light chain in blood and urine	
Imaging examination	X ray	X-ray plain film shows sparse trabecular bone and is only sensitive to bone loss of up to 30%
		DXA quantifies bone density by targeting T-values in the lumbar spine, hip, or other parts of the body
	CT	CT images provide sectional anatomy and are more sensitive to minor fractures
		Quantitative CT (QCT) measures human volumetric bone density through phantom and analysis software, with a lower false negative rate than DXA
	MRI tissue has high contrast, no radiation, and is more sensitive in displaying early changes in bone marrow	
	Nuclear medicine uses radionuclides for bone imaging to reflect bone metabolism, and monitors therapeutic efficacy through bone quantification	

Dual energy X-ray absorptiometry (DXA) and quantitative CT are commonly used bone density measurement techniques in imaging examinations. DXA calculates the average bone density per unit volume area using a computer based on the attenuation imaging of two X-rays passing through the lumbar spine, hip, and soft tissue. The DXA diagnostic criteria for osteoporosis developed by WHO are applicable to postmenopausal women, women who have undergone ovariectomy, and men over 50 years old, and are evaluated using T-values; Children, adolescents, premenopausal women, and men under 50 years old were calculated using the Z-values from the same race population database (Table 3). The volumetric bone density measured by quantitative CT is not affected by the surrounding tissues of the measurement area, and the measurement site is mainly the lumbar spine, with a higher radiation dose than DXA. The American College of Radiology proposed taking the absolute average of trabecular bone density of two lumbar vertebrae (commonly the first and second lumbar vertebrae) as the diagnostic criteria for osteoporosis, but there is no unified QCT diagnostic standard internationally. Its role in evaluating the efficacy of osteoporosis drugs and predicting the risk of osteoporotic fractures is not yet clear (Table 3).

**TABLE 3:** Diagnostic criteria for DXA and QCT osteoporosis.

Diagnosis	DXA T value^*^	QCT lumbar spine bone density value^**^
Normal	T value ≥ −1.0 SD	Volumetric BMD >120 mg/cm^3^
Low bone mass	−2.5 SD < T value < −1.0 SD	80 mg/cm^3^≤Volumetric BMD≤120 mg/cm^3^
Osteoporosis	T value ≤ −2.5 SD	Volumetric BMD≤80 mg/cm^3^
Severe osteoporosis	T value ≤ −2.5 SD,with brittle fracture	Volumetric BMD≤80 mg/cm^3^,with brittle fracture

^*^T value=(Actual measured bone density value - peak bone density value of normal individuals)/Standard deviation of peak bone density in normal individuals (SD).

^**^The specified amount of lumbar spine bone density is measured by CT, and the volume bone density of lumbar trabecular bone is measured. The average value of trabecular bone density of two lumbar vertebrae is taken.

Although DXA is currently recognized as the “gold standard” for diagnosing osteoporosis, its imaging principle determines that it is susceptible to many factors such as patient size, bone degeneration, vertebral fractures, and vascular calcification (Bolotin, 2007; Xu et al., 2019). In addition, the uneven distribution of bone minerals reduces the accuracy of bone density measurement. Moreover, DXA can only provide static bone density data and cannot reflect dynamic changes in bone metabolism, resulting in unsatisfactory performance in early diagnosis and treatment monitoring. In recent years, with the development of radionuclide imaging technology, the diagnostic methods for osteoporosis have been significantly expanded. Single photon emission computed tomography (SPECT) and positron emission tomography (PET) provide a new perspective for measuring bone mineral density. This technology not only visualizes bone microstructure by tracking tracer distribution in bone tissue but also captures dynamic bone metabolic activity. These capabilities offer new possibilities for early osteoporosis detection and treatment assessment, while providing a more comprehensive foundation for clinical decision-making (Cheng et al., 2020).

## 4 Principles and classification of radionuclide imaging

Radionuclides are unstable atomic nuclei that become more stable in the form of radiation by releasing energy (Collins ED and Ottingerj, 2003). These atomic nuclei emit radiation or particles during the decay process, such as alpha particles, beta particles, gamma rays, or X-rays, which release energy to transform into a more stable state. In the field of medical imaging, patients are injected with a radioactive tracer that emits positrons or photons during decay, which are captured by detectors and processed to display the metabolic activity of cells. In the treatment of certain diseases, especially tumors, the most commonly used radioactive particles include beta particles and alpha particles. Beta particles are electrons or positrons, while alpha particles are helium nuclei composed of two protons and two neutrons. These particles have different effects within cells, with beta particles typically used for direct internal radiation therapy, while alpha particles are used for targeted therapy due to their strong energy release and weaker penetration ability.

### 4.1 Nuclide imaging technology

The commonly used nuclear imaging techniques in clinical practice include scintillation imaging (gamma camera), positron emission tomography (PET), and single photon emission computed tomography (SPECT), each with its own characteristics (Table 4) (Reilly, 2019). A gamma camera is mainly composed of a collimator, a detector (scintillation crystal, photomultiplier tube), and a computer system. When radionuclide decay in the body and produce gamma rays, the gamma rays first pass through a collimator, which only allows gamma rays along a specific direction to pass through. The detector measures the incident gamma photons, processes the data through a computer system, and forms a planar image. PET is a functional imaging technique that uses radionuclides emitted by positrons to detect biological processes within the body. Positron emitting isotopes, such as Fluorine-18, are commonly used to bind to cells with active glucose metabolism, indicating the presence of tumors or other lesions. By detecting the gamma rays generated when positrons and electrons meet, high-resolution three-dimensional images can be generated, providing more detailed metabolic information. After the radioactive tracer is injected into the body, SPECT captures the emitted single photon signal through a detector to generate an image. Typically, radionuclides such as Technetium-99m, which emit gamma rays, are used as tracers that can bind to specific biomolecules. Through a rotating camera system, SPECT can collect data from multiple angles and ultimately generate three-dimensional images.

**TABLE 4:** Comparison of types of nuclide imaging techniques.

Property	γ camera	SPECT	PET
Imaging dimension	2D plane imaging	3D tomographic imaging	3D tomographic imaging
Resolution	Low	Medium	High
Radionuclide	single photon (^99m^Tc)	single photon (^99m^Tc)	positron (^18^F)
Imaging principle	Directly detecting gamma rays	Multi angle acquisition of single photon data for reconstruction	Positron annihilation produces gamma photons
Application scope	Initial screening of thyroid and bone imaging	Heart, bones, thyroid gland, brain	Tumors, heart, brain
Equipment complexity	Low	Medium	High
Cost	Low	Medium	High

### 4.2 Nuclides used in osteoporosis diseases

#### 4.2.1 ^99m^Tc

^99m^Tc is the most commonly used bone imaging nuclide, with a long half-life and moderate energy (Subramanian et al., 1972). It can bind to various bone targeting ligands and reflect bone reconstruction activity through its distribution in bone tissue. The ligands used for ^99m^Tc labeled bone imaging are mostly phosphates, mainly organic phosphine compounds.

In 1971, the first ^99m^Tc labeled monophosphate was developed and synthesized, which can bind with calcium ions in bones for imaging. It uses P-O-P as the skeleton and is easily hydrolyzed. From then until the early 1980s, various bisphosphonates emerged, with P-C-P as the basic skeleton, such as methoxyisobutyronitrile (MDP), hydroxymethylene diphosphate (HMDP or HDP), dicarboxypropane diphosphate (DPD), and dimethylaminomethylene diphosphate (DMAD). Among them, MDP is composed of methyl diphosphate and contains two phosphate groups in its chemical structure, which can bind with calcium ions in the bone. Therefore, ^99m^Tc MDP has high bone binding ability, especially in areas with active bone metabolism. It has high sensitivity to osteogenic lesions and metastatic lesions with osteogenic reactions, and can provide clear SPECT images. However, its soft tissue clearance is slow, and the time interval between drug injection and bone imaging is 2–3 h. It is not very sensitive to simple osteolytic lesions and metastases, and its diagnostic specificity for inflammation or tumors is also insufficient. The structure of ^99m^Tc HMDP contains diphosphate ions, which can bind with hydroxyapatite in bones. It is also a bisphosphonate, but its structure is relatively simpler compared to MDP. In a clinical study, the uptake ratio of ^99m^Tc HMDP in normal bone to soft tissue (B/S)was significantly higher than that in MDP after 2 and 3 h, but there was no significant difference between the two in terms of M/S (metastatic bone to soft tissue ratio) or M/B (bone metastatic to normal bone ratio) ratio (Delaloye et al., 1985). ^99m^Tc MDP and ^99m^Tc HMDP are currently the two main standard ligands for bone imaging. ^99m^Tc MDP is widely used for detecting bone metastases due to its high sensitivity, while ^99m^Tc HMDP performs better in image quality and normal bone uptake ratio, especially suitable for imaging situations that require shorter time intervals.

Until after 2000, with the development of technology, the two hydrogen atoms on the C atom in the structure of ordinary bisphosphonates P-C-P were replaced by one hydroxyl group and one alkyl group with an amino group, synthesizing third-generation bisphosphonate ligands with stronger coordination and intermolecular forces but lower lipophilicity. This not only increased the affinity for bone tissue but also improved the clearance rate in soft tissue. Pamiphosphonic acid alleviates pain in osteolytic bone metastases by inhibiting the dissolution of hydroxyapatite and the activity of osteoclasts. Scholars have demonstrated that ^99m^Tc pamidronate has higher uptake than ^99m^Tc MDP in rat bones (Kumar et al., 2007). Zoledronic acid is clinically used to treat hypercalcemia and multiple myeloma caused by bone metastases, as a highly osteogenic ligand to prepare bone imaging agents. Researchers in China have synthesized two novel derivatives of zoledronic acid using 1-ethylimidazole and 1-butylimidazole as raw materials, and labeled them with ^99m^Tc to generate ^99m^Tc HEIDP and ^99m^Tc HBIDP. Compared with ^99m^Tc MDP, these derivatives can be imaged earlier in rabbit bones, have more uptake in the spine and joints, and have better image quality. ^99m^Tc zoledronic acid is commonly used for imaging bone metastases, but there are also clinical applications of ^99m^Tc decay products labeled with zoledronic acid to treat osteoporosis.

With the development of clinical medicine, some new ligands have emerged to meet the needs of disease diagnosis. In addition to ^99m^Tc complex coupled bisphosphonates, ^99m^Tc complex coupled oligopeptides have also been developed as novel ^99m^Tc labeled bone seeking agents with good chemical properties and high stability, some of which have shown excellent effects in preclinical studies. Pentavalent technetium labeled dimercaptosuccinic acid (^99m^Tc (V) - DMSA) can be observed in some lung cancer metastases without ^99m^Tc MDP uptake (Sahin et al., 2000). Studies have shown that osteoclasts preferentially accumulate ^99m^Tc (V) - DMSA. Therefore, ^99m^Tc (V) - DMSA scanning can be used for imaging diagnosis of osteoclast type metastatic bone disease, which is beyond the reach of ^99m^Tc labeled bisphosphonates (Horiuchi-Suzuki et al., 2004).

#### 4.2.2 ^18^F

^18^F NaF (^18^F sodium fluoride) has a moderate half-life, can diffuse across membranes, and is quickly cleared by the kidneys (Blake et al., 2018). After a single pass, 50% is retained by bone and 30% is retained by red blood cells (Langsteger et al., 2016).

In fact, as early as the 20th century, ^18^F NaF was used in imaging research of bone diseases, but it was not until the 21st century, with the development of PET/CT, that it was widely used in clinical diagnosis of bone tumors and benign bone lesions. NaF has a high affinity for hydroxyapatite in bones. After intravenous injection, ^18^F - flows through bone tissue with blood and exchanges with OH - in the bone, accumulating in the bone tissue. The degree of uptake highly reflects local bone blood flow perfusion and bone metabolic activity (Czernin et al., 2010). Through PET/CT scanning, gamma photons generated by positron annihilation emitted by ^18^F are detected, forming metabolic images of the bone. In the early stages of osteoporosis, there may be micro damage to local bone trabeculae, and the body will initiate repair mechanisms, which can lead to increased local bone metabolism activity. Studies have shown that the absorption of ^18^F NaF by bones can reach twice that of ^99m^Tc MDP, therefore ^18^ F-NaF PET can detect bone metabolism abnormalities earlier than traditional methods such as bone density testing (Ahmed et al., 2022).

Not only that, PET/CT combined with CT can also provide quantitative CT (QCT) bone density measurement data for comparison of therapeutic efficacy before and after treatment. NaF PET/CT is a sensitive and specific imaging method for evaluating bone turnover in several skeletal regions. In addition to musculoskeletal diseases, it is also used to evaluate multiple regions, including the femoral neck, knee joint, hip joint, and spine (Zhang et al., 2020). Through whole-body PET scanning, it better presents the overall activity of osteoporosis.

Although ^99m^Tc labeled phosphates are still the most widely used bone imaging method in clinical practice due to price reasons, ^18^F NaF is more accurate in lesion localization, and with technological innovation, there will be broader application scenarios in the future as compared in Table 5.

**TABLE 5:** Characteristic comparison of^99m^Tc and^18^F.

Characteristics	^99m^Tc	^18^F
Physical property	Single Photon Emission Computed Tomography (SPECT) radionuclide, which emits a single gamma photon with an energy of 140Kev	Positron emission tomography (PET) radioactive isotopes emit positrons that undergo annihilation reactions with electrons in surrounding tissues, producing two opposite direction, 511Kev energy gamma photons
Half-life	360 min	109 min
Bone imaging ligand	^99m^Tc can label various organic phosphate compounds for bone imaging	At present, only NaF has been labeled with^18^F for bone imaging, while other bone ligands such as small molecule compounds and peptides are still in the experimental research stage
Imaging characteristics	The energy level can be effectively detected by SPECT detectors, and the attenuation in the tissue is relatively moderate, ensuring sufficient penetration depth and imaging quality	PET imaging can achieve high-precision localization and imaging by detecting the gamma photons generated by annihilation
Advantage	A suitable half-life ensures that the compound has sufficient time for distribution and metabolism in the body, allowing for imaging information to be obtained at multiple time points after a single injection. High cost-effectiveness and wide clinical use	The spatial resolution is relatively high, generally reaching 4–6 MM, and the advantage is more obvious in the imaging of fine structures. Stronger contrast can effectively distinguish between diseased tissue and normal tissue
Disadvantage	The spatial resolution is generally around 10–15 mm, slightly lower than the imaging quality of^18^F PET	The radiation energy is high, the radiation dose received by patients is relatively large, and the cost is high

## 5 The role of nuclear imaging in the diagnosis of osteoporosis

### 5.1 Bone imaging

The imaging agent labeled with radionuclides is adsorbed by hydroxyapatite crystals in the bone, and the distribution of the imaging agent in the bone is detected to reflect the activity level of bone metabolism. In patients with osteoporosis, due to accelerated bone turnover and imbalanced activity of osteoblasts and osteoclasts, bone imaging may show specific changes. Early osteoporosis may manifest as a relatively uniform distribution of whole-body bone imaging agents and a mild decrease in uptake. As the condition progresses, mild concentration of imaging agents may occur in areas with abnormally active bone metabolism, such as the vertebral body and hip, indicating local bone metabolism activity and the possibility of microfractures or bone repair processes. Radioisotope bone imaging can detect abnormal changes in bone metabolism in patients with osteoporosis at an early stage, which is helpful for early diagnosis. Especially for patients with atypical clinical manifestations and difficult to diagnose through routine examinations, valuable information can be provided. At the same time, radionuclide bone imaging can also assist in the diagnosis of other metabolic bone diseases, such as hyperparathyroidism, which causes bone symptoms similar to osteoporosis (Hain and Fogelman, 2002). However, in ^18^F NaF bone imaging, it will present systemic bone metabolism characteristic imaging features, which can avoid misdiagnosis due to the lack of specific local imaging techniques.

### 5.2 Bone quantification

Radioisotope bone density measurement is one of the core applications of radionuclides in the diagnosis of osteoporosis. It mainly relies on the bone uptake characteristics of radionuclide tracers. By measuring the radioactive isotope uptake in specific bone areas (such as lumbar spine, hip, etc*.*) and comparing it with normal reference values, the bone metabolic activity index is calculated. These indices can reflect the speed of bone turnover and are of great significance in determining the degree and progression of osteoporosis. At the same time, combining CT images can eliminate the influence of abnormal bone structure on the uptake of radionuclides and improve the accuracy of diagnosis.

^99m^Tc MDP remains the most widely used radionuclide for bone density measurement in clinical practice due to its high specificity and convenience, while SPECT is considered a more accurate bone quantification technique (Israel et al., 1998). Patients usually receive intravenous injection of ^99m^Tc MDP, followed by SPECT scanning within a few hours after injection. The distribution of radionuclides in bones is processed by a computer to form an image, and then the ROI technique (region of interest analysis) is used to compare the radioactive count ratios of each region on the image with the standard image to obtain bone density information, which can reduce the imaging of overlapping soft tissues on the measurement results. This method does not require invasive procedures and can provide an assessment of whole-body bone density, especially when evaluating key fracture risk areas such as the lumbar spine and hip.

In addition, ^99m^Tc MDP bone imaging can also reveal changes in bone microstructure, such as bone porosity and trabecular density, which can indicate changes in bone metabolism several weeks or even months earlier than X-ray plain films, and has certain clinical value for early detection of small fracture risks. During the process of fracture healing, the amount of radionuclides absorbed by the fracture site will significantly increase, and as the healing progresses, the amount gradually returns to normal.

## 6 The role of radionuclides in evaluating the therapeutic effect of osteoporosis

Efficacy evaluation is an important component of radionuclide therapy for osteoporosis. It not only helps to assess the treatment effect, but also provides a basis for adjusting subsequent treatment plans. Traditional assessment methods such as DXA bone density measurement are widely used, but they have delays in monitoring the treatment effectiveness of osteoporosis, which leads to unresponsive patients facing a sustained high risk of fractures for many years before corrective measures are taken (Reid, 2021). In contrast, radioactive nuclide imaging technology, especially quantitative evaluation indicators such as SUV value, has gradually become an important tool for efficacy evaluation because it can directly reflect bone metabolic activity. During the process of osteoporosis drug treatment, changes in bone metabolism before and after treatment can be observed through regular radionuclide bone imaging or quantitative bone SPECT/CT examination. For example, some anti-osteoporosis drugs regulate bone metabolism by inhibiting osteoclast activity or promoting osteoblast function. If the treatment is effective, bone imaging may show that bone metabolism activity gradually returns to normal, and the uptake of imaging agents decreases or tends to stabilize. The bone metabolic activity index in quantitative bone SPECT/CT examination may also decrease, indicating a slower rate of bone turnover and a gradual increase in bone density.

A study selected 10 postmenopausal female patients with osteoporosis as research subjects (Moore et al., 2012). The patients received treatment with teriparatide (20 μg/day subcutaneous injection) and underwent gamma camera two-dimensional bone scan imaging at baseline, 3 months, 18 months, and 6 months after discontinuation. Most subjects showed visual changes on bone scan images at 3 and 18 months, which disappeared after 6 months of discontinuation. Enhanced uptake was mainly observed in the skull and lower limbs. The median increase in skeletal *K*_bone_ was 22% (p = 0.004) at 3 months, 34% (p = 0.002) at 18 months, and decreased to 0.7% after 6 months of discontinuation. The changes in skull *K*_bone_ were three times greater than those in other areas. During the treatment with teriparatide, an increase in bone uptake of ^99m^Tc MDP indicates an increase in bone formation, supported by an increase in Bone turnover markers (BTM). After 6 months of discontinuation, all *K*_bone_ and BTM values returned to baseline levels.

In a study of 24 postmenopausal women with glucocorticoid induced osteoporosis treated with alendronate, the SUV value of ^18^F NaF in the lumbar spine was observed several months before changes in bone alkaline phosphatase (ALP) and bone density (BMD) occurred (Uchida et al., 2009). In another study, postmenopausal women with osteoporosis treated with levodopa received 18F fluoride PET scans of the lumbar spine at baseline and 6 months after treatment. It was found that the fluoride net plasma clearance rate (Ki), which represents osteoblast activity, significantly decreased after treatment, suggesting that ^18^F fluoride PET is a tool for evaluating the treatment of osteoporosis (Frost et al., 2003). The potential of using NaF PET/CT to observe and test lumbar spine treatment can open new doors for effective diagnosis and treatment of metabolic bone diseases.

The above research results indicate that radionuclides can not only be used for the diagnosis of osteoporosis, but also for metabolic quantification, disease assessment, and efficacy monitoring. In addition, fractures are a serious complication in patients with osteoporosis, and their risk is closely related to bone density and bone quality. Radionuclide examination can be used to measure small metabolic activities (Reilly et al., 2018). By observing changes in bone imaging or quantitative bone SPECT/CT before and after treatment, the degree of reduction in fracture risk with treatment can be evaluated. If the treatment is effective, bone imaging may show a more stable bone structure and accelerate the repair process at the fracture site, while improvements in bone density and bone metabolic activity index in quantitative bone SPECT/CT examination may also be associated with a reduced risk of fracture.

## 7 Discussion

In the diagnostic methods of osteoporosis, traditional DXA technology is relatively economical and easy to popularize in many hospitals and clinics, but it focuses on quantitatively evaluating bone density in specific areas and can only provide static bone density values. Radioisotope imaging can comprehensively evaluate the metabolic activity of the entire body’s bones and is suitable for screening potential fracture risks, such as SPECT and PET. It can not only accurately measure bone mineral density, but also reveal bone microstructure and metabolic activity, thereby improving the early diagnosis rate of osteoporosis and the accuracy of predicting fracture risk. The application of radioactive tracers such as ^99m^Tc MDP and ^18^F NaF in bone imaging has been proven to be valuable in clinical practice, but there is still room for improvement, such as improving image resolution, reducing radiation dose, and developing standardized evaluation criteria.

Radionuclides also play a crucial role in monitoring the effectiveness of drug therapy for osteoporosis. Bone ligand drugs such as phosphonates and collagen cross-linked tracers directly reflect changes in bone metabolism activity, providing a powerful tool for quantitatively evaluating drug efficacy. Through regular imaging, the evolution of bone density, bone porosity, and trabecular structure can be dynamically observed to adjust treatment plans. Like bone imaging, the application of radionuclides in drug monitoring is currently limited by challenges such as radiation dose management, standardization of image analysis, and development of new drugs.

Nevertheless, the future prospects of radionuclide technology are encouraging. The development of new radiopharmaceuticals, such as drugs targeting bone remodeling cell types, combined with more efficient imaging equipment and deep learning analysis, is expected to further improve the accuracy and practicality of monitoring. In addition, with the rapid development of radionuclides and radiopharmaceuticals in various fields such as cancer, Novel explorations in osteoporosis indications, along with the development of standardized protocols and the advancement of international consensus, will pave the way for groundbreaking advancements.
